# A Large-Scale Prospective Study on Functional Ability and Mortality Following Stroke Based on HRS Data Set: Implications for Rehabilitation

**DOI:** 10.3389/fpsyg.2022.915653

**Published:** 2022-07-11

**Authors:** Hansheng Zhu

**Affiliations:** The Independent Schools Foundation Academy, Hong Kong, China

**Keywords:** multi-domain, pre- and post-stroke, LASSO, longitudinal, ADLs, rehabilitation

## Abstract

According to the WHO, 15 million people worldwide suffer a stroke annually. Of these, 5 million die and another 5 million are left permanently disabled. Patients endure the impacts of strokes during their rehabilitation and afterward, placing economical and emotional burdens on family and community. Using data from the Health and Retirement Study (HRS) of the USA, the research performed a large-scale prospective analysis to examine how demographics, socioeconomic factors, cognition, emotion, and physical activity predict functional impairment and mortality. Multiple regression was employed to identify statistically significant variables that predict longitudinal Activities of Daily Life (ADLs). The least absolute shrinkage and selection operator (LASSO) logistic regression, a supervised machine learning approach, was deliberately chosen to obtain the subset of predictors that provide the best possible classification for the dependent variable. The LASSO regression produced a model with a fair mean Area Under the Curve (AUC) of 0.75 in predicting the risk of the patient's mortality. My findings also uncovered the important roles of BMI, mobility, muscle strength, memory, mental status, and socioeconomic status play in the long-term ADLs and survival of patients with stroke.

## 1. Introduction

A stroke is a cerebral vascular event that results in chronic dysfunction and mortality, which causes a heavy burden on the individual, family, and wider society. Understanding the functional impairments and mortality in the aftermath of stroke is, thus, pressing in providing better prevention and intervention and developing a better health care system for patients with stroke.

Due to the long-lasting impacts of stroke on emotion and cognition, one's long-term health may be impacted, causing deterioration in their ADLs. Researchers have concluded that impaired cognition and sustained depression after the stroke had a lingering effect on ADLs at 15 months post stroke (Pohjasvaara et al., 2002). Moreover, the research found that the coexistence of stroke and depression increases the chance of mortality compared to those with each condition independently (Ellis et al., 2010), which in turn corroborates the impairment to long-term health caused by cognitive and emotional variations following stroke. A study in 2006 examined how predictor variables of cognitive impairment such as demographic data and lesion characteristics correlate with the stroke-specific life quality of patients using multiple logistic and linear regression (Nys et al., 2006). At the end of the study, patients with different baseline cognitive disorders showed different levels of reduction in Quality of Life (QOL) after 6 months. Considering cognitive disorders in executive functioning, visual perception/construction, visual memory, and unilateral (visual) neglect; the strongest impact on the patient's health post-stroke is an impairment in visual perception/construction. Similarly, the QOL of stroke survivors with pathological depression is lower than that of those with marginal or no depression. Univariate analysis of stroke survivors at 4 years was performed to evaluate the impact of different variables including hospital anxiety depression scale classifications (anxiety and depression) and cognitive deficits on the health-related quality of life of patients after stroke (Haacke et al., 2006). Despite these studies showing a negative association between both depression and cognitive impairments with health-related quality of patients with post-stroke, few of them examined cognition and emotion as predictors when controlling other confounding variables.

Motor skill indicators (e.g., gross motor skills and large muscle index) are also included in the model since they are testified to be important indices of one's post-stroke health (Mercier et al., 2001). Past research has shown that lesion in the parietal lobe is the primary mechanism for motor disorders among patients with stroke (Jaywant et al., 2020). Motor deficits due to stroke can have severe impacts on one's daily activities by generating functional disabilities and affecting rehabilitation potential. Hence, the inclusion of motor skills indicators can further improve the ability of the model in predicting a patient's post-stroke ADL.

Besides patients' health and/or cognitive status, there were also studies showing existing racial and socioeconomic disparities in stroke incidence in the U.S., which also contributes to disparities in stroke mortality (Howard et al., 2011; Pinto et al., 2017). For instance, African American women had significantly higher mortality rates from stroke than White women despite advances in treatment and the management of risk factors (Kuhajda et al., 2006). A study in England also confirmed the strong impacts of socioeconomic disparities in the incidence of hospitalization for first-ever stroke and post-stroke survival. By including the demographic and socioeconomic variables in the analysis, the current study would be able to identify the extent to which different sub-populations are influenced by stroke differently in terms of long-term health.

Stroke research has highlighted treatment targets in rehabilitation (Cramer et al., 2017; Walker et al., 2017; Eng et al., 2019). However, few studies have examined multiple domains to elucidate contributors to better ADLs and greater life expectancy post-stroke. Furthermore, variables in these studies were almost exclusively limited to factors measured after the onset of the illness. Even when pre- and post-stroke factors were analyzed (Kang, 2021), the variables included were almost exclusively limited to factors in one or two specific domains.

To fill this critical gap, this study used pre- and post-factors across multiple domains to predict functional impairments and mortality.

This study employed a large data set that used a longitudinal design to carry out a series of analyses to determine: 1) the relative significance of variables before and after the report of stroke from demographics, cognitive and emotional health, motor skills, and socioeconomic characteristics in influencing a patient's activity of daily living; 2) statistical significance of variables in predicting post-stroke mortality.

## 2. Methods

**Figure 1:**
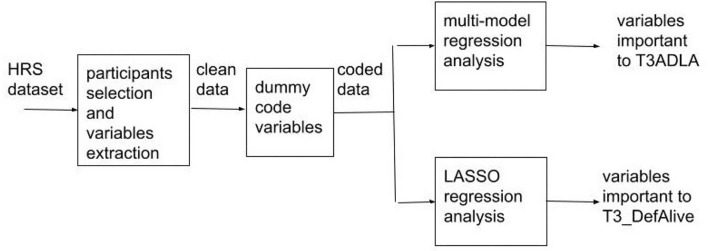
Data analysis process. T3ADLA refers to a patient's activities of daily living 4 years post-stroke, whereas T3_DefAlive is the variable definition of whether the patient is alive 4 years post-stroke.

The following Figure 1 gives a brief summary of the method.

### 2.1. Data and Participants

HRS data (HRS) is a dataset containing information from Core and Exit Interviews of the HRS, with derived variables covering a large range of topics including demographics, cognitive and emotional health, socioeconomic characteristics, and family structure. Since the data was not particularly created for stroke patients, its validity would not be affected by researcher bias.

A total of 2,342 participants (1,030 men and 1,312 women) averaging 73 years old (SD = 10.06) at the wave when they experienced stroke for the first time were selected from the data set. Multiple domains of variables were included: demographics, cognitive and emotional health, socioeconomic characteristics, and health indices.

To elucidate how pre- and post-stroke factors were related to long-term health and mortality, the researcher used a baseline floating approach, first identifying the time point (wave) when participants experienced their first stroke and then extracting variables of interest before (T0) and immediately after the onset of stroke (T1), 2 years after stroke (T2), 4 years following the stroke (T3). This approach reorganized the data into an analyzable format for the current investigation. The researcher also selected the year when the stroke occurred as a potential variant given that healthcare resources had changed dramatically over the years. Due to our interest in cognition and depression before, during, and after stroke, four waves of data for each eligible participant were included: before the report of stroke (i.e., pre-stroke), when the participant reported stroke (i.e., stroke), and following the participant's report of stroke (i.e., 2 and 4 years post-stroke). Participants who experienced a stroke in the first three waves (1992–1996) were excluded since cognition data in these waves were either different or lacked mental status measurement.

The final sample resembled the US population with 78.69% White or Caucasian, 18.27% Black or African American, and 2.90% other races. In terms of ethnicity, 5.64% of the sample were Hispanic, while the remaining 94.36% were Non-Hispanic.

### 2.2. Domains of Variables

This research took into consideration of demographic, socioeconomic, cognition and emotion, health condition, and motor skill factors when constructing our models. These variables are important predictors of post stroke health as both the prevalence rate and impacts of stroke after-care are disproportionate across different demographic groups.

Variables such as gender, race, heart problems, lung problems, high blood pressure, psychiatric problems, gross motor skills, mobility, and diabetes were dummy coded for two reasons: 1) To enable easier interpretation; 2) To enable incorporation into binary models. For race, there were three categories, White, Black, and Others. Given that few belonged to others and it is unclear what the category's composition is, we dummy coded the variables such that White is 0 and Black and others are 1. Similarly, the gender variable (GENDER) is recoded such that 1 is male and 0 is female. For medical conditions, they originally include multiple conditions. For instance, heart problem (HEART) was initially included in the following way: no heart problem as 1, heart attack as 2, coronary heart disease as 3, angina as 4, congestive heart failure as 5, and heart valve complications as 6. No diagnosed health problem was coded as 0 and health problem diagnosed as 1 in present study as only the presence of these conditions could impact ADL and mortality. Lung problem (LUNG), high blood pressure (HIBP), and psychiatric problem (PSYCH) are recoded in the same pattern. The wealth of each participant is indicated by the variable ATOTN (total non-housing assets), which reflects assets other than real estate—the financial ability of each patient—following stroke. Cognition was evaluated in the form of a total cognition score that adds the total recall and mental status indices together. These indices are obtained through the tests on immediate and delayed word recall (DLRC), the serial 7s test (SER7), backward counting from 20, object naming, and vocabulary (VOCAB) (the University of Michigan and National Institute of Aging, 2020). DLRC is a count of the number of words from the 10 or 20 words immediate recall list that were recalled correctly after a delay of about 5 min spent answering other survey questions. The Serial 7s test asks the individual to subtract 7 from the prior number, beginning with 100 for five trials. Correct subtractions are measured, and the valid scores are 0–5. Depression was measured by the 8-item version of the Center for Epidemiologic Studies Depression (CESD) scale. The index includes six “negative” indicators (depression, everything is an effort, sleep is restless, felt alone, felt sad, and could not get going) and two “positive” indicators (felt happy and enjoyed life). Finally, the variable “activities of daily living” were selected to elucidate a multi-aspect understanding of the cognition performance of patients with stroke, as physical disability can exemplify a form of cognitive impairment. We also imputed a binary variable to reflect the patient's mortality at each wave (i.e., 0 means dead and 1 means alive) based on the interview date and death date.

### 2.3. Analytic Plan

Analyses were performed in Rstudio version 1.4.1717 (R Project for Statistical Computing, 2021). The original code can be viewed on GitHub. A baseline floating approach was employed to examine cognition and depression in the context of stroke. Descriptive data analysis was performed to understand whether stroke impacts the development of depression and cognition. Multiple regression is then utilized by finding the optimal parameters for the predictions to fit a Gaussian curve. This regression analysis determines to what extent demographics, post-stroke depression, cognitive impairment, socioeconomics, and functional impairment predict the long-term health (indicated by ADLs) among patients with stroke. The model was estimated using the following formula:


(1) $$\begin{array}{c} Y_{i}=\beta_{0}+\beta_{1}X_{1}{}_{i}+\beta_{2}X_{2}{}_{i}+...+\beta_{k}X_{k}{}_{i}+\varepsilon_{i}| \end{array}$$

*Y*_*i*_ is the dependent variable, which is the patients' ADLs in this context. All the selected variables are then arranged from *X*__1_*i*_ to *X*__*k*_*i*_. To better represent the depression data, we used the statistical modeling program Mplus to classify the depression trajectories of all individual patients into meaningful subgroups, which were later included as independent variables in the models. Specifically, we constructed a multiple regression model to investigate the relationship between the patients' ADL and indicators of their emotional cognitive (e.g., depression trajectories, cognition score), socioeconomic, and functional status.

Later, the least absolute shrinkage and selection operator (LASSO)—a type of supervised machine learning approach—was used to select the most robust predictors for mortality (T3_DefAlive). The goal of LASSO regression is to obtain the subset of predictors that maximizes prediction accuracy for an outcome variable. LASSO achieves this by shrinking the coefficients of non-important predictors to zero. Given the relatively large number of predictors and relatively small sample size, traditional multiple regression analyses are underpowered to detect all effects and subject to multicollinearity and overfitting issues. LASSO, on the contrary, applies a penalization parameter that reduces regression coefficients of less important predictors to zero. This not only addresses the issues of multicollinearity and model overfitting but also improves the interpretability of the resulting statistical model (Tibshirani, 1996; McNeish, 2015). To select lambda—the optimal shrinkage parameter—for the LASSO model and obtain mean cross-validation estimates of model performance, 10-fold cross-validation (90% data set for training and 10% set for validation) with three repetitions was conducted when building the model using the caret package in R (Kuhn, 2021), following the recommendation of Kuhn and Johnson (2013). The model uses the following penalty to ridge regression:


(2) $$\begin{array}{r} SSE_{l1}=\overset{n}{\underset{i=1}{\sum }}{\left(y_{i}-{ŷ}_{i}\right)}^{2}+\lambda \overset{P}{\underset{j=1}{\sum }}|\beta_{i}| \end{array}$$

Given that splitting the data set into a single training and test set makes it difficult to accurately characterize uncertainty in prediction, especially when the sample size is small, this study used repeated 10-fold cross-validation to provide more reasonable estimates of how accurate the resultant model would be in using the same variables to predict long-term health of patients with stroke in unseen future data sets. Using the VarImp() function of the caret package, variable importance was estimated and visualized by reordering the predictor coefficients that are not shrunken to zero on a scale from 0 to 100 with the predictor of the greatest magnitude scoring 100.

## 3. Results

The selected sample included a total of 2,342 participants. Our data included evaluations of T0, T1, T2, and T3. Across the 4 assessments, 751 were deceased within 4 years post-stroke.

**Figure 2:**
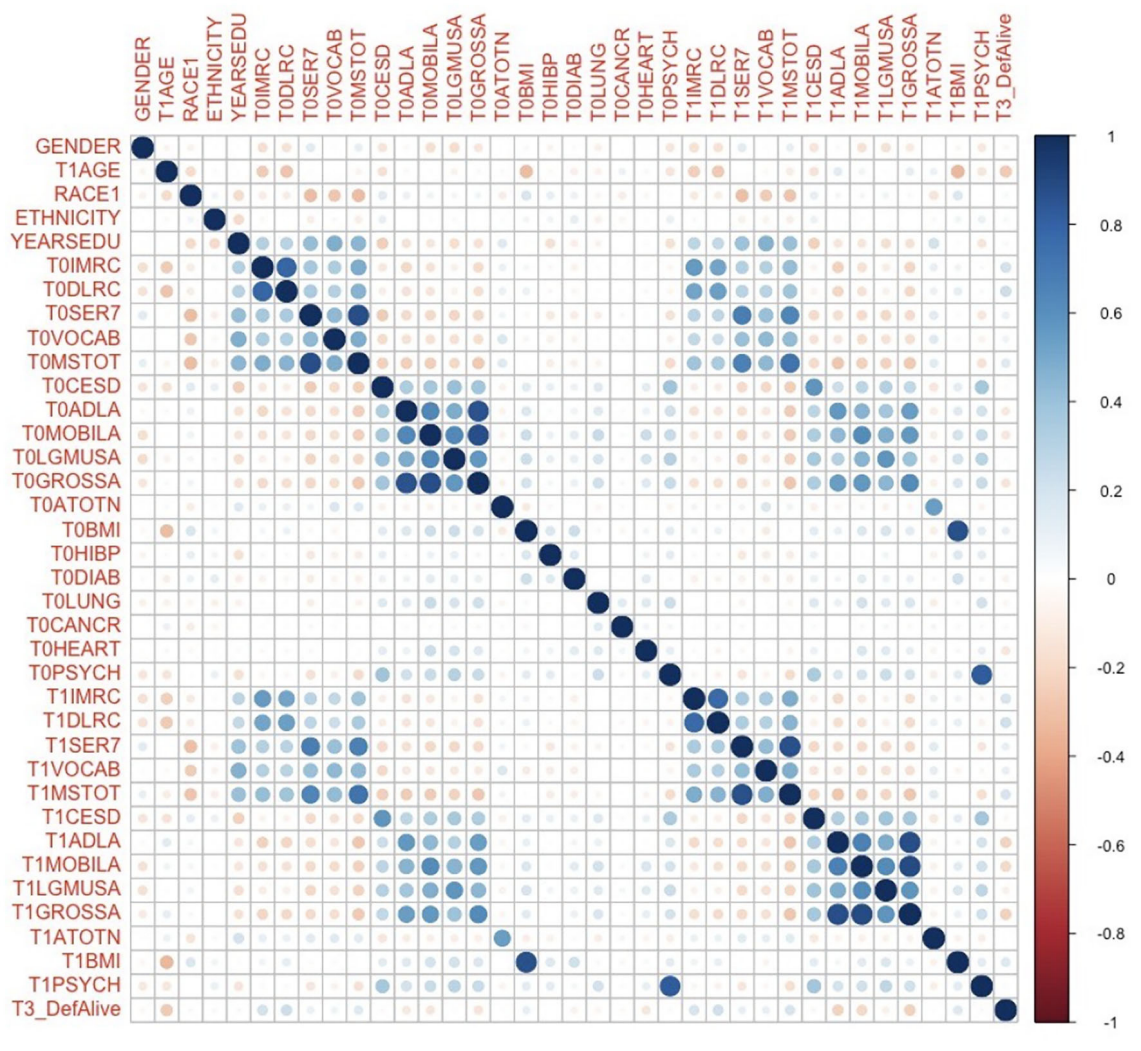
Correlation plot of variables that would be included in future regression models. T0 in the variable name means the corresponding variable before the stroke. T1 in the variable name means the corresponding variable immediately after the onset of stroke. T3 in the variable name means the corresponding variable 4 years following a stroke. YEARSEDU, YEARS of EDUcation; IMRC, IMmediate word ReCall; DLRC, DeLayed word ReCall; SER7, SERial 7s test; VOCAB, VOCABulary score; MSTOT, TOTal Mental Status summary score; CESD, The Center for Epidemiologic Studies Depression scale; ADLA, Activity of Daily Life; MOBILA, MOBILity; LGMUSA, LarGe MUScle; GROSSA, GROSS motor skills; ATOTN, TOTal Non-house Assets; BMI, Body Mass Index; HIBP, HIgh Blood Pressure; DIAB, DIABetes; PSYCH, PSYCHiatric. Refer to for the meanings of variables in detail.

Before any regression analysis, a correlation test was performed on all variables that would be included in future models to check for multicollinearity. From the correlation plot (Figure 2), it can be observed that although some pre-stroke variables are correlated with their post-stroke counterparts, most variables of different categories are not strongly correlated with each other. Therefore, future models would not encounter strong multicollinearity.

The multiple regression model in the study used demographics, pre- and post-stroke emotion, cognition, socioeconomics, and health condition indicators as independent variables to predict the dependent variable each patient's ADLs 4 years post-stroke. The summarized results shed light on the relative importance of each variable in predicting ADLs 4 years following the stroke (T3ADLA). The patients' activities of daily living right after stroke (T1ADLA) have the highest statistical significance (*P* < 0.001) and a positive association with the patient's ADLs 4 years post-stroke, meaning that higher difficulty in daily activities right after stroke onset correlates with higher difficulty in daily activities 4 years after stroke. Conversely, the pre-stroke ADLs of the patient (T0ADLA) are insignificant in predicting post-stroke ADLs at all (*P* = 0.46). Apart from T1ADLA, variables like gender, LGMUSA, MOBILA, and heart problems also have strong statistical (*P* < 0.01) significance in predicting ADLs 4 years following a stroke. Besides, the gender variable positively predicts the ADLs, which indicates that male patients tend to have larger difficulty in daily activities 4 years after stroke. Moreover, LGMUSA and MOBILA also have strong statistical significance and a positive correlation with ADLs. The heart problem variable (HEART) shares a negative correlation with the ADLs, indicating that patients with more heart issues tend to have less difficulty in daily activities 4 years after stroke. Finally, the total non-housing assets of a patient (T0ATOTN) is a marginally significant (P < 0.1) variable that has a negative correlation with the DV. In other words, the more the patient's initial asset, the less difficulty in daily activities four years after the onset of stroke.

**Figure 3:**
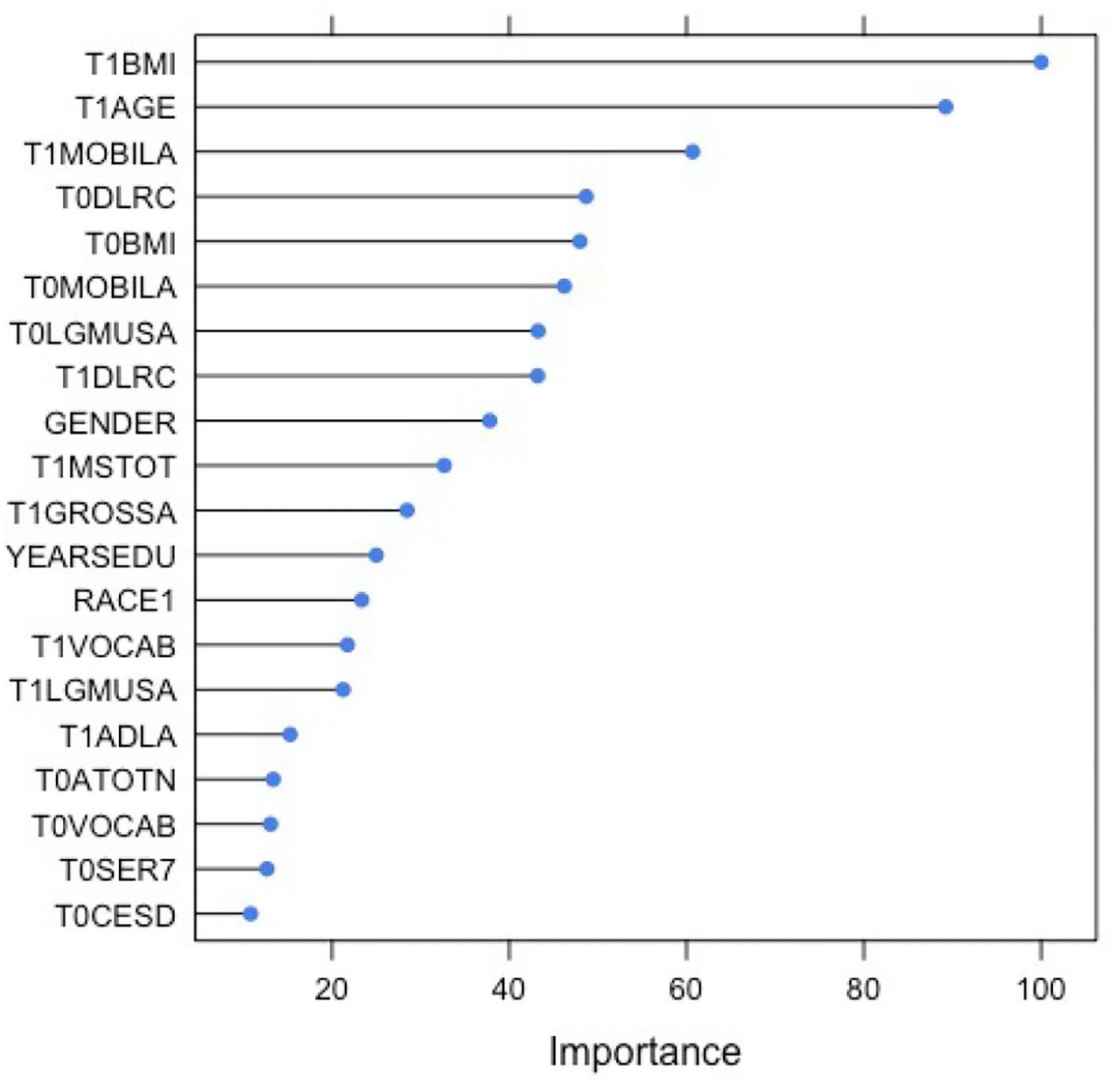
The plot of 20 most influential variables in predicting post-stroke mortality (T3_DefAlive). T0 in the variable name means the corresponding variable before the stroke. T1 in the variable name means the corresponding variable immediately after the onset of stroke. BMI, Body Mass Index; MOBILA, MOBILity; LGMUSA, LarGe MUScle; DLRC, DeLayed word ReCall; MSTOT, TOTal Mental Status summary score; GROSSA, GROSS motor skills; YEARSEDU, YEARS of EDUcation; VOCAB, VOCABulary score; ATOTN, TOTal Non-house Assets; SER7, SERial 7s test; CESD, The Center for Epidemiologic Studies Depression scale. Refer to for the meanings of variables in detail.

The least absolute shrinkage and selection operator (LASSO) regression was used to analyze the predictors for patients' mortality. LASSO penalizes less important predictors by shrinking their coefficients to 0, and can thus better address issues of multicollinearity and overfitting. Three LASSO models are specified: Model A only includes pre-stroke variables, Model B only includes post-stroke variables, and Model C includes both. The study also obtained the cross-validation estimates of model performance. The LASSO model that includes both pre- (T0) and post-stroke (T1) variables has a fair performance (0.73 < AUC < 0.78, mean AUC = 0.75) in predicting the likelihood of the patients' mortality. The performance of the model that only includes post-stroke variables is less accurate, which has a mean AUC of (0.68 < AUC < 0.72, mean AUC = 0.70) in predicting mortality. The model that only includes pre-stroke variables also has a similar mean AUC: 0.69 < AUC < 0.72, mean AUC = 0.70). Models with only pre- and post-stroke predictors exhibited comparable performance, though they were less accurate in classification than the model with both pre- and post-stroke factors. Therefore, subsequent analyses were only performed for the model with the highest accuracy. Based on the LASSO models, the study examined the relative importance of all variables included and plotted the top 20 most influential predictors of mortality (Figure 3).

According to the plot, post-stroke BMI has the most important (100%) impact on mortality. Other health-related variables like post-stroke mobility (62%) and pre-stroke large muscle index (45%) are also relatively important predictors. Additionally, cognition variables such as pre- (48%) and post-stroke (45%) delayed word recall and post-stroke mental status summary score (36%) are also predictive of the patients' mortality. Finally, demographics like age (90%) and gender (38%) are both determining factors in the prediction of post-stroke mortality.

## 4. Discussion

### 4.1. Discussion of the Present Findings

Ten factors that lead to stroke from “The Lancet” (O'Donnell et al., 2010) show: history of hypertension; current smoking; waist-to-hip ratio; diet risk score; regular physical activity; diabetes mellitus; alcohol intake; psychosocial stress and depression; cardiac causes; and the ratio of apolipoproteins B to A1. Collectively, these risk factors accounted for 88.1% of the PAR for all strokes. On our list, T0HIBP (corresponds to hypertension, first above) is not significant to ADLs. The reason could be that blood pressure is effectively controlled after a stroke. Smoking is not on our list, perhaps for the same reason. BMI (waist-to-hip ratio, the third above) is the most significant factor influencing ADLs because ADLs worsen BMI and then restrain ADLs.

Patients' ADLs right after a stroke have the highest statistical significance in predicting their ADLs 4 years following a stroke. On the contrary, patients' ADLs before stroke are insignificant in predicting ADLs of patients 4 years following a stroke. This discrepancy in predictability demonstrates how stroke may alter one's ADLs from its original trajectory, making pre-stroke ADLs information less predictive. In addition, the majority of statistically significant predictors for ADL are physical health indices. Baseline LGMUSA measures the strength of large muscles before the stroke, which strongly correlates with one's physical health. Its statistical significance in turn highlights the positive influence of a high pre- stroke muscle strength on ADL in the aftermath of a stroke. Similarly, a patient's mobility immediately after stroke onset also positively correlates with their long-term ADLs. Hence, an improvement in one's post-stroke mobility holds the potential to improve their ADL. Surprisingly, the presence of pre-stroke heart problems is associated with better post-stroke ADLs. This may be attributed to the possibility that patients who originally have heart problems may already have experience in cardiac rehabilitation, which reduces their difficulties in adapting to post-stroke daily living (Ellis et al., 2010). Finally, patients' pre-stroke total non-housing assets are a marginally significant socioeconomic factor for better ADLs. In other words, patients with higher socioeconomic status before stroke are likely to perform activities of daily living better 4 years following a stroke.

Based on the LASSO model constructed in this research, the classification of post-stroke mortality vs. survival using pre- and post-stroke demographic, socioeconomic, cognition, emotion, and physical health indicators was fairly accurate. Although the LASSO model includes 37 variables, its AUC value is less than 0.75. This shows that the relationship between mortality and pre- and post-stroke variables included in the study is unlikely to be linear. Additionally, the AUC of the model that includes both pre- and post-stroke variables (0.73) is not significantly higher than that of models with only post-stroke variables (0.70). Hence, we can infer that pre-stroke variables are important but are unable to significantly improve the performance of the LASSO model. The variables' importance plot (based on the LASSO regression) shows the relative importance of different factors in predicting one's post-stroke mortality. These results suggest that physical health indicators such as BMI, MOBILA, and LGMUSA have the strongest impact on one's mortality. Cognition variables including mental status summary score and pre- and post-stroke delayed word recall are also significant predictors of a patient's mortality. Finally, post-stroke mortality more likely happens to men and older adults, which demonstrates the importance of demographics in influencing mortality.

### 4.2. Limitations and Future Directions

Although the study is overall successful in demonstrating the relative importance of multi-domain pre- and post-stroke variables in predicting functional abilities and mortality among a large sample of patients with stroke, there are also several limitations: 1) Using the prediction of patients' ADLs for 4 years after stroke as the criteria for the importance of pre- and post-stroke variables presents bias as only survivors can be investigated. Nevertheless, this limitation is already partially addressed in the study as both ADLs and mortality are employed as indicators of long-term health. Hence, the effect of pre- and post-stroke variables on stroke patients who did not survive is also identified. 2) The relatively low AUC values of our LASSO models may result from other likely important variables that cannot be investigated given the nature of this pre-collected, open-access data set; 3) As a large-scale longitudinal study assessing multi-domain factors of a large sample, the measures included in this study are limited. For instance, the only emotion indicator included in the data set is depression. Without emotion-related measures such as anxiety and post-traumatic stress, the regression analysis performed is unlikely to reflect the full picture of whether and how emotion predicts long-term health; Future directions can be suggested based on the current limitations:

1) To further improve the accuracy of the models in informing the prognosis of stroke, deep learning can be applied to summarize more patterns of the variables. Subsequently, the models will not just be limited to linear regression, and the variables will not necessarily need to be dichotomized.
2) Future studies examining other potential neurocognitive predictors such as anxiety may provide additional information to findings from the current investigation and shed light on long term risk and resilience among patients with stroke.
3) To identify more individual differences of patients with stroke that could lead to different health consequences, future datasets that include both quantitative and qualitative data may improve existing rehabilitative treatment to improve post stroke prognosis.

## 5. Conclusion

The study examined the relative significance of pre- and post-stroke demographic, socioeconomic, cognition, emotion, and physical health indicators as predictors of functional impairments and mortality among a large sample of patients with stroke. We arrived at the conclusion that: 1) activities of daily living, mobility just after stroke, heart problem, large muscle index before the stroke, and gender are the significant variables influencing a patient's activities of daily living. 2) BMI, AGE, mobility just after stroke, BMI mobility, and large muscle index before stroke are the most important factors in predicting post-stroke mortality.

The results of the study are insightful in showing that:

1) Given the important roles BMI, mobility (MOBILA), and large muscle index (LGMUSA) play in long-term ADLs and mortality, it is highly desirable for patients with stroke to incorporate diet, exercise, and behavioral rehabilitation to improve their movement skills in order to maintain long-term functional capacity and reduce the likelihood of mortality.
2) Given the importance of pre-stroke delayed memory and mobility in predicting mortality, monitoring and improving memory and movement skills among the geriatric population have the potential to reduce one's likelihood of poor functional outcomes after a stroke occurs.
3) Due to the importance of post-stroke delayed memory and mental status in reducing the likelihood of mortality, cognitive remediation should also be implemented in improving one's post-stroke health;
4) The positive correlation between the socioeconomic status and the patients' ADLs raises the issue of extra caring and financial support required for patients with stroke in lower-SES families.

## Author's Note

The code used to perform the analysis can be found online at https://github.com/4818002/Longitudinal_Stroke_Analysis.

## Data Availability Statement

Publicly available datasets were analyzed in this study. This data can be found here: https://hrsdata.isr.umich.edu/data-products/2018-rand-hrs-fat-file.

## Ethics Statement

Ethical review and approval was not required for the study on human participants in accordance with the local legislation and institutional requirements. Written informed consent for participation was not required for this study in accordance with the national legislation and the institutional requirements. Written informed consent was obtained from the individual(s) for the publication of any potentially identifiable images or data included in this article.

## Author Contributions

HZ contributed to the conception and design of the study, organized the database, performed the statistical analysis, and wrote this manuscript.

## Conflict of Interest

The author declares that the research was conducted in the absence of any commercial or financial relationships that could be construed as a potential conflict of interest.

## Publisher's Note

All claims expressed in this article are solely those of the authors and do not necessarily represent those of their affiliated organizations, or those of the publisher, the editors and the reviewers. Any product that may be evaluated in this article, or claim that may be made by its manufacturer, is not guaranteed or endorsed by the publisher.

## References

[B1] Cramer A. S. C. Wolf A. S. L. Harold P. Adams J. (2017). Stroke recovery and rehabilitation research: issues, opportunities, and the nih strokenet. Stroke 48, 813–819. 10.1161/STROKEAHA.116.015501 28174324 PMC5330812

[B2] Ellis A. C. Zhao A. Y. Egede A. L. E. (2010). Depression and increased risk of death in adults with stroke. J. Psychosom. Res. 68, 545–551. 10.1016/j.jpsychores.2009.11.006 20488271 PMC2874721

[B3] Eng A. J. J. Bird A. M.-L. Godecke A. E. (2019). Moving stroke rehabilitation research evidence into clinical practice: consensus-based core recommendations from the stroke recovery and rehabilitation roundtable. Int. J. Stroke 14, 766–773. 10.1177/1747493019873597 31564224

[B4] Haacke A. C. Althaus A. A. Spottke A. A. (2006). Long-term outcome after stroke evaluating health-related quality of life using utility measurements. Stroke 37, 193–198. 10.1161/01.STR.0000196990.69412.fb 16339458

[B5] Howard A. V. J. Kleindorfer A. D. O. Judd A. S. E. (2011). Disparities in stroke incidence contributing to disparities in stroke mortality. Ann. Neurol. 69, 619–627. 10.1002/ana.22385 21416498 PMC3595534

[B6] Jaywant A. A. DelPonte A. L. Kanellopoulos A. D. (2020). The structural and functional neuroanatomy of post-stroke depression and executive dysfunction: a review of neuroimaging findings and implications for treatment. J. Geriatr. Psychiatry Neurol. 35, 3–11. 10.1177/0891988720968270 33073704

[B7] Kang C. (2021). Predictors of post-stroke cognition among geriatric patients: The role of demographics, pre-stroke cognition, and trajectories of depression. Front. Psychol. 12, 717817. 10.3389/fpsyg.2021.717817 34381407 PMC8349975

[B8] Kuhajda A. M. C. Cornell A. C. E. Nell A. J. (2006). Training community health workers to reduce health disparities in alabama's black belt the pine apple heart disease and stroke project. Family Commun. Health 29, 89–102. 10.1097/00003727-200604000-00005 16552287

[B9] Kuhn M. (2021). caret: Classification and Regression Training. R package version 6.0-88.

[B10] Kuhn M. Johnson K. (2013). Applied Predictive Modeling. 10.1007/978-1-4614-6849-3

[B11] McNeish D. M. (2015). Using lasso for predictor selection and to assuage overfitting: A method long overlooked in behavioral sciences. Multivariate Behav. Res. 50, 471–484. 10.1080/00273171.2015.1036965 26610247

[B12] Mercier A. L. Audet A. T. Hébert A. R. (2001). Impact of motor, cognitive, and perceptual disorders on ability to perform activities of daily living after stroke. Stroke 32, 2602–2608. 10.1161/hs1101.098154 11692024

[B13] Nys A. G. Zandvoort A. M. der Worp A. H. (2006). Early cognitive impairment predicts long-term depressive symptoms and quality of life after stroke. J. Neurol Sci. 247, 149–156. 10.1016/j.jns.2006.04.005 16716359

[B14] O'Donnell A. M. J. Xavier A. D. Liu A. L. (2010). Risk factors for ischaemic and intracerebral haemorrhagic stroke in 22 countries (the interstroke study): a case-control study. Lancet. 376, 112–123. 10.1016/S0140-6736(10)60834-3 20561675

[B15] Pinto A. C. B. Velez A. F. G. S. Lopes A. F. (2017). Ssri and motor recovery in stroke: reestablishment of inhibitory neural network tonus. Front. Neurosci. 11, 637. 10.3389/fnins.2017.00637 29200995 PMC5696576

[B16] Pohjasvaara A. T. Vataja R. Leppävuori A. Kaste M, Erkinjuntti, T. (2002). Cognitive functions and depression as predictors of poor outcome 15 months after stroke. Cerebrovasc. Dis. 14, 228–233. 10.1159/000065667 12403956

[B17] Tibshirani R. (1996). Regression shrinkage and selection via the lasso. J. Royal Statist. Soc. Ser. B. 58, 267–288.

[B18] Walker A. M. F. Hoffmann A. T. C. Brady A. M. C. (2017). Improving the development, monitoring and reporting of stroke rehabilitation research: consensus-based core recommendations from the stroke recovery and rehabilitation roundtable. Int. J. Stroke 12, 472–479. 10.1177/1747493017711815 28697706

